# Prevalence of substance consumption using the Alcohol, Smoking, and Substance Involvement Screening Test (ASSIST) and the Quit Using Drugs Intervention Trial (QUIT): brief intervention at Tijuana and Los Angeles health centers

**DOI:** 10.1186/1940-0640-8-S1-A51

**Published:** 2013-09-04

**Authors:** Guillermina Natera-Rey, Miriam Arroyo, Lillian Gelberg, Ronald Andersen

**Affiliations:** 1 National Institute of Psychiatry, Ramón de la Fuente Muñiz, Mexico City, Mexico; 2 University of California, Los Angeles, USA

## 

There is evidence of the growth of substance consumption throughout the world. However, there continues to be a broad gap between the scope of the problem and the use of preventive and treatment services. This situation is particularly acute near the border between Mexico and the United States, where drug use and availability are greater. Funding was obtained in both countries to conduct a feasibility study about the early identification of consumption at primary health care centers and to test the Quit Using Drugs Intervention Trial (QUIT) of brief intervention. Early identification and brief intervention have proven to be cost-effective and to encourage timely care and reduce the care gap. Men and women aged 18+ (approximately 1000 in each country) will be screened with the Alcohol, Smoking and Substance Involvement Screening Test (ASSIST). Risky users (about 65 participants in each country) will be enrolled and randomized to either QUIT or a control condition. An electronic material management application (EMMA) and “talking touch-screen” wireless tablets will be used for screening, randomization, and data monitoring. The main results of the implementation phase are the creation of a binational research team; consolidation of the research design; adaptation of instruments and strategies of the ASSIST-QUIT intervention and the EMMA system; technology exchange; and identification of areas requiring cultural adaptation. An ethnographic approach to health centers in Tijuana, Mexico and Los Angeles was used to identify the differences and similarities between centers. Comparisons of the prevalence of drug consumption among participants in both countries will be presented. The main contribution is the identification of the challenges, scope, and cultural differences in the implementation of a program for early detection and treatment of drug consumption in primary health care centers in the border region. The results will underpin new research to apply this model among similar populations.

